# Diagnostic accuracy of contrast-enhanced ultrasound for microvascular invasion of hepatocellular carcinoma: A protocol for systematic review and meta-analysis

**DOI:** 10.1097/MD.0000000000029083

**Published:** 2022-04-15

**Authors:** Yanli Chen, Haiyan Qiao, Zhaoan Lian, Chunlin Li, Yi Xiang

**Affiliations:** a Department of Ultrasonography, The Central Hospital of Enshi Tujia and Miao Autonomous Prefecture, Enshi, Hubei, China; b Department of Pathology, The Central Hospital of Enshi Tujia and Miao Autonomous Prefecture, Enshi, Hubei, China; c Department of Gynaecology and Obstetrics, The Central Hospital of Enshi Tujia and Miao Autonomous Prefecture, Enshi, Hubei, China; d Radiology Catheterization Room, The Central Hospital of Enshi Tujia and Miao Autonomous Prefecture, Enshi, Hubei, China.

**Keywords:** contrast-enhanced ultrasound, diagnosis, hepatocellular carcinoma, meta-analysis, microvascular invasion, protocol

## Abstract

**Background:**

Microvascular invasion is an independent risk factor for the recurrence of hepatocellular carcinoma (HCC). Early detection and timely treatment can reduce the recurrence and prolong the overall survival of HCC. Contrast-enhanced ultrasound (CEUS) has been validated for their predictive potential of microvascular invasion in HCC patients, although the conclusion remains controversial. Therefore, a meta-analysis is necessary to be conducted to validate the diagnostic value of CEUS for microvascular invasion in HCC, thus supporting guideline development and clinical practice.

**Methods:**

Relevant studies reporting the potential diagnostic value of CEUS for microvascular invasion in HCC patients published before February 2022 will be searched in the PubMed, EMBASE, Cochrane Library, and Web of Science. Data will be extracted by 2 researchers independently. The risk of bias will be assessed by the Quality Assessment of Diagnostic Accuracy Studies-2. All of the above statistical analysis will be carried out with Stata 14.0.

**Results:**

The results of this study will be published in a peer-reviewed journal.

**Conclusion:**

This study will comprehensively evaluate the diagnostic accuracy of CEUS for microvascular invasion in HCC patients, thus providing high-quality evidence to support clinical practice and guideline development.

## 1 Introduction

Hepatocellular carcinoma (HCC) is one of the most-common malignancies.^[1,2]^ Although many treatment options are available to HCC patients, the incidence of postoperative recurrence and metastasis remains high.^[3,4]^ Microvascular invasion is one of the independent risk factors for poor prognosis of HCC, which is an important reference for clinicians to select the optimal treatment and assess the risk of recurrence and metastasis.^[5–7]^ Currently, pathological examination is the main diagnostic method for HCC, but imaging and serological examinations are also useful in predicting microvascular invasion of HCC.^[8–10]^

Ultrasonography is the most commonly used imaging method for early screening of HCC due to the features of simple operations, nonradioactive and noninvasive procedures.^[11]^ Contrast-enhanced ultrasound (CEUS) is a noninvasive diagnostic technique that has been increasingly used in the diagnosis of HCC.^[12–14]^ Hepatic artery blood supply is the main lesion of HCC. Microvascular circulation changes when HCC lesions are invaded by microvessels, which can be dynamically examined by CEUS.^[15–17]^ Therefore, CEUS is of great importance in predicting microvascular invasion of HCC.

Currently, a consensus on the recognized guidelines for CEUS in predicting microvascular invasion of HCC is lacked due to the subjective determination of operators and various models of machines. Controversial findings have been reported among various studies.^[18–21]^ Therefore, a meta-analysis is necessary to verify the diagnostic value of CEUS for microvascular invasion of HCC and to support guideline development and clinical practice. In this study, we will evaluate the diagnostic value of CEUS for microvascular invasion of HCC by conducting a meta-analysis, thus obtaining scientific conclusions for clinical practice.

## 2 Methods

### 2.1 Study registration

The protocol of the systematic review has been registered on Open Science Framework (registration number: DOI 10.17605/OSF.IO/ZC95V). This diagnostic meta-analysis was conducted in accordance with the Preferred Reporting Items for a Systematic Review and Meta-analysis of Diagnostic Test Accuracy Studies (PRISMA-DTA) statement.^[22]^

### 2.2 Inclusion criteria

#### 2.2.1 Type of studies

Studies reporting the diagnostic value of CEUS in predicting the microvascular invasion of HCC.

#### 2.2.2 Type of participants

Patients with HCC over 18 years old.

#### 2.2.3 Type of examination

HCC patients receive CEUS examination.

#### 2.2.4 Reference standards

Pathology or cytology is the gold standard for the diagnosis of microvascular invasion of HCC.

#### 2.2.5 Outcome measurements

Outcomes include the pooled sensitivity (SEN), specificity (SPE), positive likelihood ratio, negative likelihood ratio, diagnostic odds ratio, area under the curve, and their 95% confidence intervals.

### 2.3 Exclusion criteria

- Literatures with incomplete data;
- Republished literatures;
- Case reports, reviews, cellular experiments, or animal experiments.

### 2.4 Data sources and search strategy

Relevant studies reporting the potential diagnostic value of CEUS for microvascular invasion in HCC patients published before February 2022 will be searched in the PubMed, EMBASE, Cochrane Library, and Web of Science databases. In addition, references of included literatures will be manually reviewed. Literature retrieval strategies in the PubMed were shown in Table 1.

**Table 1:** Search strategy (PubMed).

Number	Search terms
#1	Carcinoma, Hepatocellular[MeSH]
#2	Hepatocellular Carcinoma[Title/Abstract]
#3	Hepatoma[Title/Abstract]
#4	Liver Cancer, Adult[Title/Abstract]
#5	Liver Cell Carcinoma[Title/Abstract]
#6	Liver Cell Carcinoma, Adult[Title/Abstract]
#7	Adult Liver Cancer[Title/Abstract]
#8	Adult Liver Cancers[Title/Abstract]
#9	Cancer, Adult Liver[Title/Abstract]
#10	Cancers, Adult Liver[Title/Abstract]
#11	Carcinoma, Liver Cell[Title/Abstract]
#12	Carcinomas, Hepatocellular[Title/Abstract]
#13	Carcinomas, Liver Cell[Title/Abstract]
#14	Cell Carcinoma, Liver[Title/Abstract]
#15	Cell Carcinomas, Liver[Title/Abstract]
#16	Hepatocellular Carcinomas[Title/Abstract]
#17	Hepatomas[Title/Abstract]
#18	Liver Cancers, Adult[Title/Abstract]
#19	Liver Cell Carcinomas[Title/Abstract]
#20	or/1-19
#21	Microvascular invasion[Title/Abstract]
#22	Vascular invasion[Title/Abstract]
#23	or/21-22
#24	Contrast-enhanced ultrasound [Title/Abstract]
#25	Contrast-enhanced ultrasonography[Title/Abstract]
#26	Ultrasonography[Title/Abstract]
#27	CEUS[Title/Abstract]
#28	or/24-27
#29	Diagnosis∗[Title/Abstract]
#30	Sensitivity[Title/Abstract]
#31	Specificity[Title/Abstract]
#32	ROC curve[Title/Abstract]
#33	or/29-32
#34	#20 and #23 and #28 and #33

### 2.5 Data collection and analysis

#### 2.5.1 Study selection and data extraction

**Figure 1:**
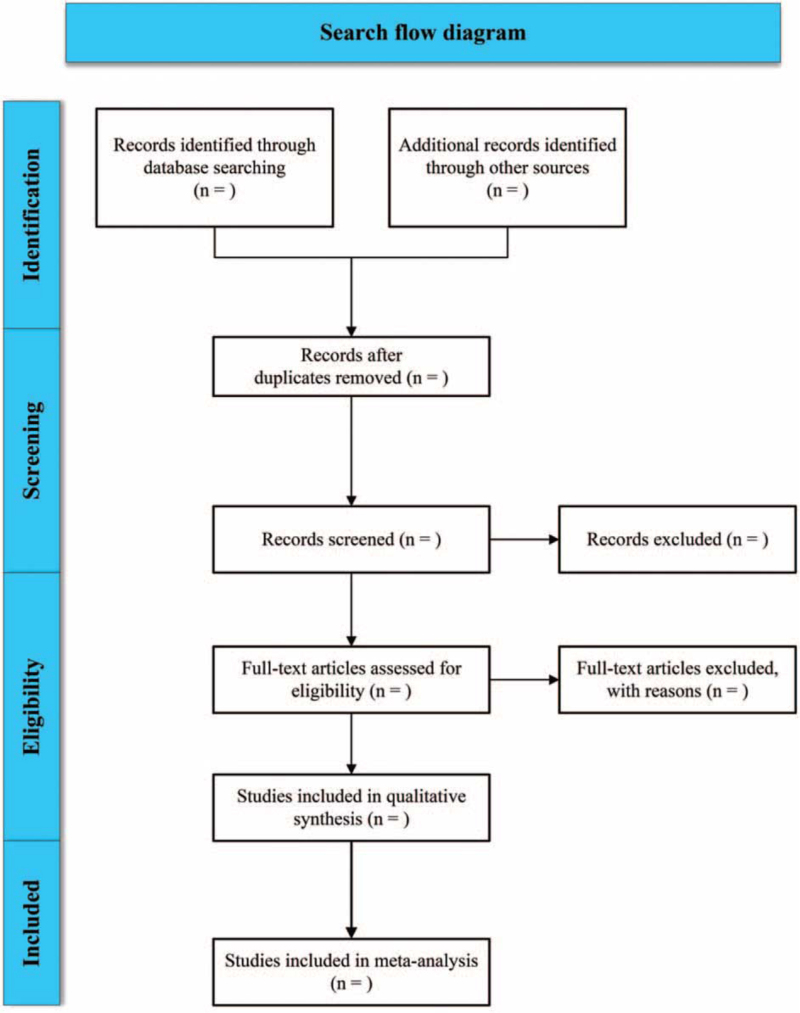
Flow diagram showing literature filtration process.

Two investigators will be independently responsible for literature screening, data extraction and cross-check. Any disagreement will be solved by the third researcher. Missing information will be obtained by contacting the author. Relevant literatures will be first screened by reading the title and abstract. After excluding obviously irrelevant literatures, the full-text will be further reviewed. The screening flow chart of this study was demonstrated in Figure 1. A self-made data extraction form will be used to extract information, including the first author, publication year, regions, types of studies, age and number of participants, diagnostic threshold, outcome indicators, etc.

### 2.6 Quality assessment

The methodological quality of the included studies will be assessed following Quality Assessment of Diagnostic Accuracy Studies-2 criteria,^[23]^ consisting of 4 key domains (patient selection, index test, reference standard, and flow and timing).

### 2.7 Statistical analysis

Statistical analysis will be performed with Stata 14.0 (Stata Corp, College Station, TX). The presence of a threshold effect will be determined by obtaining the Spearman correlation coefficient and observing whether the summary receiver operating characteristic curve has a “shoulder-arm” distribution. The heterogeneity of the non-threshold effects will be tested by the Chi-square test. *I*^2^ ≤ 50% and *P* ≥ .1 indicates no heterogeneity, and the data will be analyzed using a fixed-effects model; otherwise, a random-effects model will be adopted. The pooled SEN, SPE, positive likelihood ratio, negative likelihood ratio, diagnosis odds ratio, and their 95% confidence interval will be calculated. In addition, the pooled diagnostic value of CEUS in predicting the microvascular invasion of HCC through the summary receiver operating characteristic and area under the curve will be tested.

### 2.8 Subgroup analysis

Subgroup analyses based on the ethnicity, and follow-up time will be conducted.

### 2.9 Sensitivity analysis

Goodness of fit and bivariate normal analysis will be performed to test the stability of the results.

### 2.10 Publication bias

The publication bias will be determined by Deeks’ funnel plot asymmetry test.

### 2.11 Ethics and dissemination

The content of this study does not involve moral approval or ethical review and it will be presented in print or at relevant conferences.

## 3 Discussion

Vascular invasion is one of the most important events triggering HCC recurrence and metastasis, and microvascular invasion is one of the vascular invasion modes, which can only be detected by microscopic examination and serves as a marker for predicting early tumor metastasis.^[24,25]^ Therefore, accurate prediction of microvascular invasion in HCC is of great significance for selecting the optimal treatment and assessing the prognosis of HCC.

This is the first meta-analysis to comprehensively retrieve and summarize the evidence for CEUS to predict microvascular invasion of HCC. The results of this review will provide a clinical basis for predicting microvascular invasion of HCC and a novel direction for future explorations.

## Author contributions

**Data curation:** Haiyan Qiao.

**Formal analysis:** Zhaoan Lian.

**Methodology:** Chunlin Li.

**Project administration:** Yi Xiang.

**Supervision:** Yi Xiang.

**Validation:** Chunlin Li.

**Visualization and software:** Haiyan Qiao.

**Writing – original draft:** Yanli Chen and Yi Xiang.

**Writing – review & editing:** Yanli Chen and Yi Xiang.

## References

[1] Oshi M Kim TH Tokumaru Y. Enhanced DNA repair pathway is associated with cell proliferation and worse survival in hepatocellular carcinoma (HCC). Cancers 2021;13:01–15. 10.3390/cancers13020323 PMC7830462 33477315

[2] Xiao S Huang S Yang J. Overexpression of GIHCG is associated with a poor prognosis and immune infiltration in hepatocellular carcinoma. OncoTargets Ther 2020;13:11607–19. 10.2147/OTT.S271966 PMC7670177 33209037

[3] Zhou Z Qi L Mo Q. Effect of surgical margin on postoperative prognosis in patients with solitary hepatocellular carcinoma: a propensity score matching analysis. J Cancer 2021;12:4455–62. 34149909 10.7150/jca.57896 PMC8210564

[4] Dong P Hu J Peng X Zhang C Yang W. Advances in preoperative prediction of microvascular invasion in hepatocellular carcinoma. J MuDanJiang Med Univ 2022;43:119–21.

[5] Yanhan W Lianfang L Hao L. Effect of microvascular invasion on the prognosis in hepatocellular carcinoma and analysis of related risk factors: a two-center study. Front Surg 2021;8:01–7. 10.3389/fsurg.2021.733343 PMC8637807 34869551

[6] Chen ZH Zhang XP Wang H. Effect of microvascular invasion on the postoperative long-term prognosis of solitary small HCC: a systematic review and meta-analysis. HPB 2019;21:935–44. 30871805 10.1016/j.hpb.2019.02.003

[7] Chen Y Liu H Zhang J. Prognostic value and predication model of microvascular invasion in patients with intrahepatic cholangiocarcinoma: a multicenter study from China. BMC Cancer 2021;21:1299. 34863147 10.1186/s12885-021-09035-5 PMC8645153

[8] Çelebi F Görmez A Ilgun AS Tokat Y Balci NC. The value of 18F-FDG PET/MRI in prediction of microvascular invasion in hepatocellular carcinoma. Eur J Radiol 2022;149:110196. 35144118 10.1016/j.ejrad.2022.110196

[9] Wang J Ding ZW Chen K Liu YZ Li N Hu MG. A predictive and prognostic model for hepatocellular carcinoma with microvascular invasion based TCGA database genomics. BMC Cancer 2021;21:1337. 34911488 10.1186/s12885-021-09047-1 PMC8675478

[10] Yu YX Hu CH Wang XM. Value of the application of enhanced CT radiomics and machine learning in preoperative prediction of microvascular invasion in hepatocellular carcinoma. Zhonghua Yi Xue Za Zhi 2021;101:1239–45. 34865392 10.3760/cma.j.cn112137-20200820-02425

[11] Jiang Y Zhang M Zhu Y Zhu D. Diagnostic role of contrast-enhanced ultrasonography versus conventional B-mode ultrasonography in cirrhotic patients with early hepatocellular carcinoma: a retrospective study. J Gastrointest Oncol 2021;12:2403–11. 34790401 10.21037/jgo-21-611 PMC8576229

[12] Kang HJ Lee JM Yoon JH Han JK. Role of contrast-enhanced ultrasound as a second-line diagnostic modality in noninvasive diagnostic algorithms for hepatocellular carcinoma. Korean J Radiol 2021;22:354–65. 33236540 10.3348/kjr.2020.0973 PMC7909851

[13] Dong Y Wang WP Mao F. Imaging features of fibrolamellar hepatocellular carcinoma with contrast-enhanced ultrasound. Ultraschall Med 2021;42:306–13. 32102105 10.1055/a-1110-7124

[14] Wang F Numata K Okada M. Comparison of Sonazoid contrast-enhanced ultrasound and gadolinium-ethoxybenzyl-diethylenetriamine pentaacetic acid MRI for the histological diagnosis of hepatocellular carcinoma. Quant Imaging Med Surg 2021;11:2521–40. 34079721 10.21037/qims-20-685 PMC8107308

[15] Lin M Zhang X Chen Z Xie X Kuang M. Application of quantitative contrast-enhanced ultrasound perfusion analysis in preoperative evaluation of differentiation of hepatocellular carcinoma. Chin J Hepatic Surg 2019;8:353–7.

[16] Yang L Gu Y Lu J Liu H. Contrast-enhanced ultrasonography versus contrast-enhanced MRI in the evaluation of therapeutic effect of TACE for HCC: comparison of application value. J Interv Radiol 2019;28:682–6.

[17] Zhao S Liu L Chen M. Correlation between the contrast-enhanced ultrasonography of hepatocellular carcinoma and microvascular invasion. J Guangxi Med Univ 2019;36:886–9.

[18] Xuan Z Wu N Li C Liu Y. Application of contrast-enhanced ultrasound in the pathological grading and prognosis prediction of hepatocellular carcinoma. Transl Cancer Res 2021;10:4106–15. 35116708 10.21037/tcr-21-1264 PMC8799228

[19] Zhang D Wei Q Wu GG. Preoperative prediction of microvascular invasion in patients with hepatocellular carcinoma based on radiomics nomogram using contrast-enhanced ultrasound. Front Oncol 2021;11:709339. 34557410 10.3389/fonc.2021.709339 PMC8453164

[20] Zhou H Sun J Jiang T. A nomogram based on combining clinical features and contrast enhanced ultrasound LI-RADS improves prediction of microvascular invasion in hepatocellular carcinoma. Front Oncol 2021;11:699290. 34307168 10.3389/fonc.2021.699290 PMC8297520

[21] Zhu W Qing X Yan F Luo Y Li Y Zhou X. Can the contrast-enhanced ultrasound washout rate be used to predict microvascular invasion in hepatocellular carcinoma? Ultrasound Med Biol 2017;43:1571–80. 28502665 10.1016/j.ultrasmedbio.2017.04.003

[22] Salameh JP Bossuyt PM McGrath TA. Preferred reporting items for systematic review and meta-analysis of diagnostic test accuracy studies (PRISMA-DTA): explanation, elaboration, and checklist. BMJ (Clin Res ed) 2020;370:m2632. 10.1136/bmj.m2632 32816740

[23] Diao W Su D Cao Y Jia Z. The diagnostic accuracy of O-(2-18F-fluoroethyl)-L-tyrosine parameters for the differentiation of brain tumour progression from treatment-related changes. Nucl Med Commun 2022;43:350–8. 35102078 10.1097/MNM.0000000000001524

[24] Zhang EL Cheng Q Huang ZY Dong W. Revisiting surgical strategies for hepatocellular carcinoma with microvascular invasion. Front Oncol 2021;11:691354. 34123861 10.3389/fonc.2021.691354 PMC8190326

[25] Zhang K Tao C Wu F Siqin T Wu J Rong W. Establishment and evaluation of a predictive model for early postoperative recurrence of hepatocellular carcinoma in patients with microvascular invasion. Int J Gen Med 2021;14:2259–74. 34113155 10.2147/IJGM.S303896 PMC8184236

